# Early Childhood Neurodevelopmental Outcomes After Early Infant Invasive Group B Streptococcal Infection in Uganda

**DOI:** 10.1093/ofid/ofae602

**Published:** 2025-03-10

**Authors:** Samantha Sadoo, Carol Nanyunja, Mary Kyohere, Hannah G Davies, Valerie Tusubira, Cleophas Komugisha, Joseph Peacock, Margaret Sewegaba, Philippa Musoke, Musa Sekikubo, Kirsty Le Doare, Cally J Tann, PROGRESS Study Author Group, Abdelmajid Djennad, Agnes Nyamaizi, Agnes Ssali, Alexander Amone, Amusa Wamawobe, Annettee Nakimuli, Caitlin Farley, Carol Nanyunja, Christine Najuka, Cleophas Komugisha, Dan R Shelley, Edward A R Portal, Ellie Duckworth, Emilie Karafillakis, Geraldine O’Hara, Godfrey Matovu, Hannah G Davies, Janet Seeley, Joseph Peacock, Juliet Nsimire Sendagala, Katie Cowie, Kirsty Le Doare, Konstantinos Karampatsas, Lauren Hookham, Madeleine Cochet, Margaret Sewegaba, Mary Kyohere, Maxensia Owor, Melanie Etti, Merryn Voysey, Moses Musooko, Musa Sekikubo, Owen B Spiller, Patience Atuhaire, Paul T Heath, Philippa Musoke, Phiona Nalubega, Pooja Ravji, Richard Katungye, Ritah Namugumya, Rosalin Parks, Rose Azuba, Sam Kipyeko, Simon Beach, Stephen Bentley, Tim Old, Tobius Mutabazi, Valerie Tusubira, Vicki Chalker

**Affiliations:** London School of Hygiene and Tropical Medicine, London, UK; University College London Hospital, London, UK; London School of Hygiene and Tropical Medicine, London, UK; Medical Research Council/Uganda Virus Research Institute and London School of Hygiene & Tropical Medicine Uganda Research Unit, Entebbe, Uganda; Institute of Infection and Immunity, St George's, University of London, London, UK; Makerere University—Johns Hopkins University Research Collaboration (MUJHU), Kampala, Uganda; London School of Hygiene and Tropical Medicine, London, UK; Institute of Infection and Immunity, St George's, University of London, London, UK; Makerere University—Johns Hopkins University Research Collaboration (MUJHU), Kampala, Uganda; Makerere University—Johns Hopkins University Research Collaboration (MUJHU), Kampala, Uganda; Institute of Infection and Immunity, St George's, University of London, London, UK; Kawempe National Referral Hospital, Kampala, Uganda; Makerere University—Johns Hopkins University Research Collaboration (MUJHU), Kampala, Uganda; Department of Obstetrics and Gynaecology, Makerere University, Kampala, Uganda; Medical Research Council/Uganda Virus Research Institute and London School of Hygiene & Tropical Medicine Uganda Research Unit, Entebbe, Uganda; Institute of Infection and Immunity, St George's, University of London, London, UK; Makerere University—Johns Hopkins University Research Collaboration (MUJHU), Kampala, Uganda; London School of Hygiene and Tropical Medicine, London, UK; University College London Hospital, London, UK; Medical Research Council/Uganda Virus Research Institute and London School of Hygiene & Tropical Medicine Uganda Research Unit, Entebbe, Uganda

**Keywords:** infant sepsis, group B *Streptococcus*, growth, neurodevelopment, Uganda

## Abstract

**Background:**

Group B streptococcal (GBS) sepsis during infancy is a leading cause of child mortality and an important contributor to long-term neurodisability. Data on outcomes among invasive GBS infection survivors in low- and middle-income countries are limited. We present 2-year neurodevelopment and growth outcomes after GBS sepsis in Uganda.

**Methods:**

Participants were infants with culture-proven GBS sepsis <3 months of age and a gestationally matched comparison cohort of infants who did not have GBS sepsis in Kampala, Uganda. Neurodevelopmental impairment up to 24 months (corrected age) was assessed using the Bayley Scales of Infant Development and Hammersmith Infant Neurological Examination. Weight, height, mid-upper arm circumference, and occipito-frontal circumference were measured.

**Results:**

Neurodevelopmental outcome data were available for 16 survivors of GBS sepsis and 59 comparison children. Among survivors of GBS sepsis, cognitive and language scores were lower (median difference [interquartile range], −5 [−10 to 0] and −8 [−15 to −2], respectively). Moderate to severe neurodevelopmental impairment occurred in 31% (5/16) in the GBS cohort compared with 8.5% (5/59) in the non-GBS cohort. Three children with neurodevelopmental impairment had cerebral palsy (bilateral spasticity), and 2 had global developmental delay without cerebral palsy. GBS sepsis survivors were more likely to have undernutrition compared with comparison children (25% vs 10%), largely due to severe undernutrition among those with cerebral palsy.

**Conclusions:**

In this Sub-Saharan African population, survivors of infant GBS sepsis were more likely to have impaired neurodevelopmental and growth outcomes compared with children who did not have GBS sepsis. GBS sepsis survivors should be included in long-term follow-up programs to monitor for neurodevelopmental difficulties and initiate early referrals to support services.

Sepsis is a leading cause of child morbidity and mortality globally, with the greatest burden in low- and middle-income countries (LMICs) and the highest regional burden in Sub-Saharan Africa [1]. Group B *Streptococcus* (GBS; *Streptococcus agalactiae*) is the most common cause of sepsis and meningitis among infants <3 months of age [2]. While significant gains have been made in reducing child mortality globally, there has been less focus on longer-term morbidity and disability outcomes from common early childhood conditions.

The Global Strategy for Women's, Children's, and Adolescents’ Health supports the need for every newborn to not only “survive” but also “thrive” and reach their full neurocognitive and developmental potential [3]. Globally, there are an estimated 53 million children under 5 years of age living with developmental disabilities, and this has remained unchanged over recent decades [4]. The Nurturing Care Framework emphasizes the need for tiered services to meet children's needs [5], including targeted support for at-risk children and specialized services for developmental disabilities; however, data on the prevalence of adverse early childhood outcomes among at-risk children in LMIC settings are limited.

Colonization with GBS is estimated to affect between 20% and 35% of pregnant women, and of those born to colonized mothers, early-onset invasive GBS disease occurs in ∼1% [6]. While intrapartum antibiotics prophylaxis has reduced the incidence of early-onset disease (<7 days), no prophylaxis strategy exists for late onset (7 days–3 months) [7]. Each year, an estimated 40 000 survivors of GBS sepsis develop moderate to severe neurodevelopmental impairment (NDI) [8]. Up to half of GBS meningitis survivors have NDI, one-fifth moderate to severe [9].

Neurodevelopmental sequelae after sepsis include cerebral palsy, global developmental delay, visual and hearing impairment, and seizures [10]. These not only impact the child's health, functioning, and life chances, but also have substantial social, emotional, and financial impacts on children, families, and societies, particularly in LMICs [11]. Quality data are lacking on long-term neurodevelopmental outcomes after early childhood infections, particularly from LMICs. Studies are often limited by a follow-up period of <1 year of age, when NDI may be missed, and a diagnosis and classification of cerebral palsy may not be possible.

The primary aim of this study was to assess early childhood outcomes, specifically neurodevelopmental and growth outcomes, among survivors of GBS sepsis during early infancy in Uganda.

This paper forms part of a supplement based on the PROGRESS study. The Progressing Group B Streptococcal Vaccines (PROGRESS) study aimed to describe the causes of infectious mortality and morbidity in pregnancy and neonates, as well as the seroepidemiology of group B streptococcal infection—the major cause of neonatal sepsis worldwide—in Kampala, Uganda. Detailed information regarding the PROGRESS research protocol and results has been published separately [12].

## METHODS

### Study Design

This study was a prospective cohort comparison study of neurodevelopmental outcomes at 24 months among infants affected with invasive GBS sepsis (case cohort), with a matched comparison cohort of infants without GBS sepsis (comparison cohort). Case cohort infants were recruited through the PROGRESS study, a prospective observational cohort study conducted between November 2019 and April 2021 in 2 hospitals in Kampala, Uganda (Mulago National Referral Hospital and Kawempe National Referral Hospital), aiming to evaluate the feasibility and acceptability of undertaking large-scale seroepidemiology surveillance of GBS in Uganda [12]. Comparison cohort infants were recruited through the PREPARE study, comprising a prospective surveillance cohort of pregnant women and following mothers and infants up to 14 weeks, in preparation for GBS maternal vaccination trials [13].

### Setting

Uganda is a low-income country situated in East Africa, ranking 176th out of 193 countries for GDP per capita [14]. Uganda has a population of around 43 million, of whom 1.6 million live in the capital city, Kampala. The neonatal mortality rate is 19 deaths per 1000 live births, and the infant mortality rate is 31 per 1000 live births [15].

### Participants

The eligibility criteria for the PROGRESS study included neonates and young infants (<90 days of age) presenting to either study site with signs or symptoms consistent with infection (sepsis, meningitis, or pneumonia), who subsequently had a positive blood culture for GBS [12]. Any gestational age was eligible; gestational age was measured using the Ballard score or, where not available, early antenatal ultrasound/the mother's reported date of last menstrual period. For those whose gestational ages were unknown, for the purposes of neurodevelopmental assessment scoring, they were estimated from birthweight using INTERGROWTH-21st charts, which are based on normative values from children in Sub-Saharan Africa [16].

The comparison cohort of noncases was recruited from the PREPARE study [13, 17] in a 3:1 ratio of noncases to cases. Eligibility for the comparison cohort included infants who remained healthy with no hospital admissions up to 90 days of age and were matched for gestational age to the cases [13]. Prospective written informed consent was obtained from caregivers.

### Diagnosis of GBS

Blood culture samples were taken under sterile precautions and processed at the Makerere University Clinical Microbiology Laboratory (MUCML), which is accredited by the College of American Pathologists. Samples were incubated for 5 days in an automatic BACTEC machine (BACTEC 9050, 9120 Becton Dickinson, Plymouth, UK, or FX40 Becton Dickinson, Franklin Lakes, NJ, USA). A positive blood culture was defined as being positive for GBS growth.

### Neurodevelopmental Follow-up

Neurodevelopmental assessments were performed at MNRH children's outpatient clinic by a trained and experienced medical clinical officer, blind to clinical history but not to study arm. The Bayley Scales of Infant Development, 3rd Edition (BSID-III), were used to assess development at 6, 12, and 24 months of age in the cases cohort and at 24 months only in the comparison cohort [18]. BSID-III is a widely used neurodevelopment tool for children age 1–42 months, assessing neurodevelopment across 5 domains: cognition, receptive language, expressive language, fine motor, and gross motor. BSID-III scores are based on age at assessment (corrected for prematurity, <36 weeks’ gestation), with standard cutoff thresholds for NDI derived from high-income country populations: 85–89 for mild NDI, 70–84 moderate, and <70 severe [19].

All children were examined neurologically using a standardized scorable assessment, the Hammersmith Infant Neurological Examination (HINE), which has been validated as a predictor of motor outcome in diverse cohorts, at 3, 6, 12, and 24 months of age [20, 21]. A score of ≥67 has been shown to be predictive of independent walking at 2 years among high-risk infants [22]. Cerebral palsy was diagnosed and classified according to the Surveillance of Cerebral Palsy in Europe hierarchical classification [23], and severity was assigned using the Gross Motor Function Classification System for Cerebral Palsy (GMFCS) [24]. For our primary analysis, moderate to severe NDI was defined as any of the following: a BSID-III score of 2 SDs below the mean derived from the comparison cohort in any domain, a HINE score <67, and/or diagnosis of cerebral palsy.

Visual and hearing assessments were conducted according to HINE standardized procedures [18]. Visual assessment assessed fixing and following of a clear black/white target; hearing assessed response to an auditory stimulus (rattle held behind the visual range of both sides). Impairment was categorized as mild, moderate, or severe, according to HINE scoring criteria [20].

### Assessment of Growth Outcomes

Anthropometric measurements were taken at each follow-up time point for both cohorts using standardized procedures: weight (using SECA354 electronic scales, Hamburg, Germany), mid-upper arm circumference (MUAC; MUAC tape measure), height (SECA210 measuring mat), and occipito-frontal head circumference (paper tape measure). World Health Organization growth charts were used to assign Z-scores for each measurement, with a Z-score <−2 indicating moderately to severely impaired growth outcomes for weight for age (undernutrition), height (stunting), weight for height (wasting), and occipito-frontal head circumference (microcephaly) [25]. For MUAC, 11.5–12.5 mm defined moderate acute malnutrition, and <11.5 mm defined severe acute malnutrition [25]. A structured interview was conducted among caregivers to investigate caregiver concerns regarding health, growth, and development, including questions about seizures and other neurological problems.

### Statistical Analysis

Descriptive statistics were reported using medians and interquartile ranges (IQRs) for continuous variables and frequencies and percentages for categorical variables. Associations were reported using the chi-square/Fisher exact test. A comparison of median values was performed using the Wilcoxon rank-sum test, with median differences calculated using the generalized Hodges-Lehmann approach for continuous variables and risk ratios calculated for categorical variables with 95% CIs. A *P* value <.05 was considered statistically significant. STATA (version 17.1) was used for all statistical analyses.

## RESULTS

### Participants

**Figure 1.**
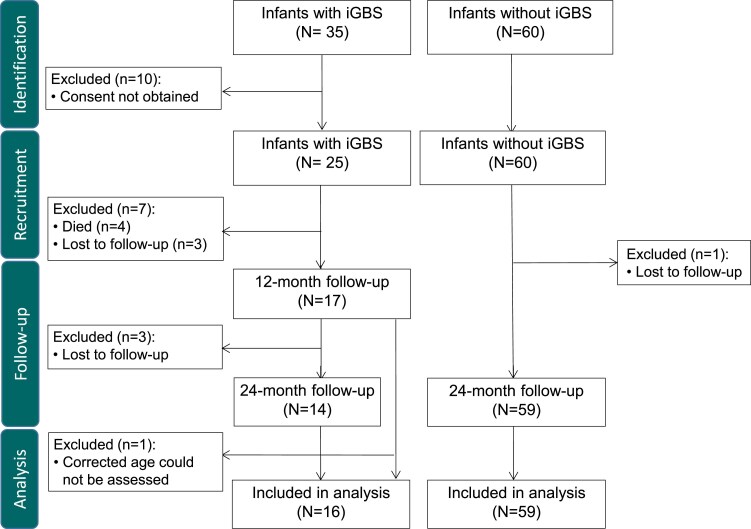
Flow of participants. Abbreviation: iGBS, invasive group B *Streptococcus*.

The flow of participants through the study is shown in Figure 1. Between April 2019 and November 2021, 25 infants were recruited to the cases cohort, and between November 2020 and May 2021, 60 infants were recruited to the comparison cohort. Of 25 participants in the cases cohort, 16 survivors (76.2%) had neurodevelopmental outcome data and were included in the analysis. All 16 were participants who had GBS growth confirmed on blood culture. Of these, 14 underwent final assessment at 24 months, and 2 at 12 months. Of those who did not have neurodevelopmental outcome data, 4 died (in the neonatal period), 3 were lost to follow-up, 1 withdrew and 1 was excluded as they attended follow-up to 12 months but were unable to be scored as corrected gestational age could not be ascertained. Of those recruited to the non–invasive GBS (iGBS) comparison cohort, 59 (98.3%) attended neurodevelopmental follow-up at 24 months and were included in the analysis.

### Baseline Characteristics

Baseline and early clinical characteristics of the case and comparison cohorts are shown in Table 1. Mothers of iGBS survivors were more likely to be of younger age, primiparous, HIV positive, to have completed up to primary education only, and to have a normal vaginal delivery when compared with mothers in the comparison cohort (none significant). Infants with iGBS sepsis were significantly more likely to need resuscitation at the time of birth. No differences were seen in sex, gestation, or birthweight.

**Table 1.** Baseline and Clinical Characteristics Among Invasive GBS Sepsis Survivors and Comparison Non-GBS Cohorts

Clinical Characteristics	iGBS Cases Cohort (n = 16)	Non-iGBS Comparison Cohort (n = 59)	*P* Value^b^
Maternal factors			
Education, ≤primary level	25 (4)	12 (7)	.19
Maternal age			
Median [IQR], y	23 [20–28.5]	26 [23–31]	.08
<20 y	19 (3)	5 (3)	.11
Primiparity	50 (8)	25 (15)	.06
HIV positive	19 (3)	8 (5)	.24
C-section	13 (2)	24 (14)	.33
Neonatal factors			
Sex, male	50 (8)	49 (29)	.95
Gestational age^a^			
Median [IQR], y	40 [37.5–40]	39 [38–40]	.22
≤37 wk	13 (2)	17 (10)	.67
Birthweight			
Median [IQR], g	3150 [2560–3450]	2900 [2600–3500]	.90
≤2500 g	19 (3)	20 (12)	.89
Resuscitation required	25 (4)	5 (3)	.02

Data shown are median [IQR] for continuous data and % (No.) for categorical data.

Abbreviations: GBS, group B *Streptococcus*; iGBS, invasive group B *Streptococcus*; IQR, interquartile range; LMP, last menstrual period.

^a^Missing data on gestational age for 10 children in the iGBS cohort; of those with known gestation, 5 were determined from Ballard score and 1 from LMP. In the non-iGBS cohort, 33 were determined from Ballard score, 16 from LMP.

^b^Wilcoxon rank-sum test for continuous variables, chi-square test for categorical variables.

### Neurodevelopmental Outcomes

The neurodevelopmental outcomes among both groups are shown in Table 2.

**Table 2.** Neurodevelopmental and Nutritional Outcomes Between Survivors of GBS Sepsis and the Comparison Cohort at Two Years of Age

…	iGBS Cases Cohort (n = 16)	Non-iGBS Comparison Cohort (n = 59)	Median Difference [95% CI]^a^	*P* Value^b^
Developmental and neurology outcomes				
Bayley Scales of Infant Development–III				
Median (IQR) [range]	…	…	…	…
Cognitive	80 (77.5–92.5) [55–95]	85 (80–90) [75–95]	−5 [−10 to 0]	.10
Language	78 (72.5–86) [47–97]	86 (83–91) [74–100]	−8 [−15 to −2]	.003
Motor	86.5 (79–89.5) [46–94]	85 (82–88) [76–94]	0 [−6 to +3]	1.00
Moderate–severe neurodevelopmental impairment by domain^c^				
Cognitive	31.3 (5)	6.8 (4)	RR, 4.6 [1.4–15.2]	.02
Language	25.0 (4)	0.0 (0)	-	.001
Motor	18.8 (3)	1.7 (1)	RR, 11.1 [1.2–99.3]	.03
Any domain	31.3 (5)	8.5 (5)	RR, 3.7 [1.2–11.2]	.03
Hammersmith Infant Neurological Exam				
Median (IQR) [range]	74 (70.5–76.8) [18.5–78]	73 (72–74) [67–77]	+1 [−2 to +3.5]	.30
<67	18.8 (3)	0 (0)	-	.008
Cerebral palsy	18.8 (3)	0 (0)	…	.008
Severe (GMFCS level 3–5)	18.8 (3)	0 (0)	-	.008
Global developmental delay (no CP)	12.5 (2)	0 (0)	-	.04
Visual impairment	18.8 (3)	0 (0)	-	.008
Hearing impairment	18.8 (3)	0 (0)	-	.008
Childhood seizure disorder	0 (0)	0 (0)	-	-
Any moderate–severe NDI^d^	31.3 (5)	8.5 (5)	RR, 3.7 [1.2–11.2]	.03
Occipito-frontal circumference, cm				
Median [IQR]	46.5 [45.5–48]	47 [46–48]	−0.5 [−1.7 to +0.5]	.24
Microcephaly (Z-score <−2)	6.3 (1)	3.4 (2)	RR, 1.6 [0.3–8.4]	.52
Severe microcephaly (Z-score <−3)	6.3 (1)	1.7 (1)	RR, 3.7 [0.24–55.8]	.38
Nutritional outcomes				
Undernutrition (weight for age)				
Mod–severe (Z-score <−2)	25.0 (4)	10.2 (6)	RR, 2.7 [0.9–5.4]	.21
Severe (Z-score <−3)	18.8 (3)	1.7 (1)	RR, 11.1 [1.2–99.3]	.03
Wasting (weight for height),				
Mod–severe (Z-score <−2)	12.5 (2)	6.8 (4)	RR, 1.6 [0.5–5.6]	.60
Severe (Z-score <−3)	12.5 (2)	0 (0)	-	.04
Stunting (height for age)				
Mod–severe (Z-score <−2)	25.0 (4)	16.9 (10)	RR, 1.5 [0.6–3.8]	.48
Severe (Z-score <−3)	12.5 (2)	1.7 (1)	RR, 3.4 [1.4–8.7]	.11
Mid-upper arm circumference				
Median [IQR], mm	14 [13–15]	15 [14–15.5]	−1 [−2 to 0]	.02
Mod–severe undernutrition (<125 mm)	18.8 (3)	0 (0)	-	.008
Severe undernutrition (<115 mm)	12.5 (2)	0 (0)	-	.04

Data shown are median [IQR] for continuous data and % (No.) for categorical data.

Abbreviations: BSID-III, Bayley Scales of Infant Development, 3rd Edition; CP, cerebral palsy; GBS, group B *Streptococcus*; GMFCS, Gross Motor Function Classification System; HINE, Hammersmith Infant Neurological Examination; iGBS, invasive group B *Streptococcus*; IQR, interquartile range; NDI, neurodevelopmental impairment; RR, risk ratio.

^a^Generalized Hodges-Lehmann median differences for continuous data and RR for categorical data for the iGBS cohort using the comparison cohort as the reference group.

^b^Wilcoxon rank-sum test for continuous variables, Fisher exact test for categorical variables.

^c^Moderate–severe NDI defined as a BSID-III score greater than 2 SDs below the mean of the comparison cohort, in any BSID-III domain of the comparison cohort (cognitive score <77, language <74, motor <78).

^d^Any NDI defined as any of BSID-III domain score ≥2 SDs below the mean of the comparison cohort, HINE <67, and/or GMFCS level 3–5.

^e^Missing data for 2 participants in the iGBS cohort for growth medians as they were measured at 12 months (did not attend at 24 months).

The iGBS case cohort had lower median scores compared with the comparison cohort for cognitive (median difference [IQR], −5 [−10 to 0]) and language (median difference [IQR], −8 [−15 to −2]); there was no difference in motor scores. Using standard BSID-III cutoff scores, the comparison cohort had a very high proportion of moderate NDI (59.3%) (Supplementary Information A); therefore, we determined NDI cutoff scores based on SDs below the mean in the comparison cohort for each domain: ≥1 SD below the mean defining no NDI, −1 to −2 SDs for mild NDI, −2 to −3 SDs for moderate NDI, and <−3SDs for severe NDI (z-score <−2 defining moderate–severe NDI) (Supplementary Information B). Using revised cutoff scores, those in the case cohort remained more likely to have moderate–severe NDI than those in the non-iGBS cohort (*P* = .03) (Table 2). In the case cohort, 31.3% (5/16) had moderate–severe NDI in at least 1 domain; the majority (3/5) had severe NDI. In the comparison cohort, 8.5% (5/59) had moderate NDI; none had severe NDI. Examining scores by domain, one-third of iGBS survivors had moderate–severe impairment in the cognitive and language domains, and one-quarter had moderate–severe motor impairment.

### Growth Outcomes

Table 2 shows growth outcomes in both the case and comparison cohorts. Twenty-five percent of the case cohort had moderate–severe undernutrition compared with 10% in the comparison cohort according to weight for age, and 20% compared with 0% according to MUAC. iGBS survivors were also more likely to have moderate–severe wasting (13% vs 7%) and stunting (25% vs 17%). Of the 5 iGBS survivors with NDI, all 4 had undernutrition, indicated by weight for age (3 were severe), and of these, 3 also had low MUAC scores (2 severe). Two (12.5%) in the iGBS cohort had bulbar palsy, indicated by drooling on clinical assessment.

### Description of Neurodevelopmental Outcomes After GBS Sepsis

Table 3 details the neurodevelopmental and growth outcomes of all iGBS survivors. Of those classified as having NDI, 3 (50%) had bilateral spastic cerebral palsy with global developmental delay, with very low scores across all BSID-III domains and the HINE. Two (33%) had moderate global developmental delay without cerebral palsy, with low scores across all BSID-III domains but normal HINE. Four (25%) had mild global developmental delay without cerebral palsy, with mild impairment in cognitive and language domains but normal motor scores. Two (33%) had isolated impairment in the cognitive and language domains only. Three (50%) had visual impairment and hearing impairment, 1 with severe visual impairment bilaterally; all 3 also had cerebral palsy. None had seizures reported by their caregiver at 24 months. One (6%) had severe microcephaly.

**Table 3.** Description of Neurodevelopment and Growth Outcomes for Survivors of Sepsis at 24 Months (n = 16)

No.	Age Assessed, mo	HINE Total	Tone Abnormality	Bulbar Palsy	BSID-III Cognitive	BSID-III Language	BSID-III Motor	GMFCS Level 3–5	Visually/Hearing Impaired	Type of NDI	Micro Cephaly^a^	Weight-for-Age Z-score	Mod–Severe Undernutrition (WAZ)^b^	Mod–Severe Undernutrition (MUAC)^c^
1	12	71	None	No	95	86	88	No	None	None	No	4.39	No	No
2	24	78	None	No	85	86	85	No	None	None	No	0.7	No	No
3	24	78	None	No	90	97	94	No	None	None	No	−0.56	No	No
4	24	77	None	No	95	94	88	No	None	None	No	1.25	No	No
5	12	72	None	No	95	89	94	No	None	None	No	−3.06	Severe	No
6	24	78	None	No	80	86	88	No	None	Mild cognitive impairment	No	−1.63	No	No
7	24	74	None	No	95	79	85	No	None	Mild language impairment	No	−1.61	No	No
8	24	76	None	No	80	79	88	No	None	Mild GDD without CP	No	0.63	No	No
9	23	73.5	None	No	80	74	85	No	None	Mild GDD without CP	No	−0.26	No	No
10	24	76.5	None	No	80	77	94	No	None	Mild GDD without CP	No	−0.24	No	No
11	24	76	None	No	80	74	91	No	None	Mild GDD without CP	No	0.7	No	No
12	24	70	None	No	75	77	79	No	None	Moderate GDD without CP	No	−2.01	Mod–severe	Mod–severe
13	23	74	Upper limb hypotonia	No	75	71	79	No	None	Moderate GDD without CP	No	−0.67	No	No
14	25	20	Bilateral spasticity	Yes	55	47	46	Level 5	Mod VI Mod HI	Bilateral spastic CP	No	−4.88	Severe	Severe
15	24	18.5	Bilateral spasticity	Yes	55	47	46	Level 5	Severe VI Mod HI	Bilateral spastic CP	Severe	−3.06	Severe	No
16	25	25	Bilateral spasticity	No	55	47	46	Level 5	Mild VI Mild HI	Bilateral spastic CP	No	−6.15	Severe	Severe

Abbreviations: BSID-III, Bayley Scales of Infant and Toddler Development, 3rd Edition; CP, cerebral palsy; GDD, global developmental delay; GMFCS, Gross Motor Function Classification System; HI, hearing impairment; HINE, Hammersmith Infant Neurological Examination; Mod, moderate; MUAC, mid-upper arm circumference; NDI, neurodevelopmental impairment; OFC, occipito-frontal head circumference; VI, visual impairment; WAZ, weight-for-age Z-score.

^a^Defined as OFC Z-score <−2 SDs (moderate) or <−3 SDs (severe).

^b^Defined as weight-for-age Z-score <−2 SDs (moderate) or <−3 SDs (severe).

^c^Defined as MUAC <125 mm (moderate) or <115 mm (severe).

## DISCUSSION

Our paper reports some of the only data on early childhood neurodevelopmental outcomes after GBS sepsis among children in Uganda. We found that iGBS survivors were more likely to have neurodevelopmental difficulties and adverse nutritional outcomes when compared with non-iGBS children. Among iGBS survivors, around a third had neurodevelopmental impairment, largely bilateral spastic cerebral palsy or moderate global developmental disability without cerebral palsy. Among those with cerebral palsy, all had severe undernutrition and some degree of visual and hearing impairment, with substantial long-term implications for the child and family.

A systematic review in 2017 identified 5 studies reporting neurodevelopmental outcomes in iGBS survivors, including among infants with GBS meningitis; a meta-analysis could not be performed for survivors of GBS sepsis due to limited data available [9]. Two cohorts included were unpublished: a South African cohort (median year of data collection, 2014) and a UK cohort (2015) reported moderate–severe NDI in 7.1% (5/70) and 1.6% (1/61), respectively. The remaining 3 identified studies were small historical cohorts from the 1970s in high-income countries (HICs), showing no, or very low, prevalence of NDI among survivors, perhaps due to lower rates of survival at that time [26, 27].

More recently, 2 from LMICs reported neurodevelopmental outcomes in survivors of iGBS, which were more consistent with our findings: A Mozambique study (2000–2019) found that 13% (5/39) had moderate–severe NDI, 5% (2) had severe hearing impairment, and 3% (1) had mild visual impairment [28]. However, children <5 years were assessed using the Malawi developmental assessment tool, which is primarily an early child development screening tool rather than a comprehensive assessment. A South African study found that 13.9% (6/43) of GBS sepsis/meningitis survivors had moderate–severe NDI at 5–8 years of age, compared with 4.3% [5] in the comparison cohort [29]. Two other LMIC studies reporting neurodevelopmental outcomes in iGBS survivors were a small Indian study (2006–2018), which found conversely a lower proportion of iGBS survivors with moderate–severe NDI (1/35, 2.9%) compared with the comparison cohort (3/65, 4.6%) using standardized assessments at 1–14 years [30] and a multicountry LMIC study that assessed emotional and behavioral outcomes in Argentina, India, Kenya, Mozambique, and South Africa using the Child Behaviour Checklist at 18 months–17 years [31]; in 106 school-aged children, emotional behavioral problem scores were significantly higher in iGBS survivors than comparators, though in preschool-aged children there was no difference [31].

Population-level studies reporting neurodevelopmental outcomes after iGBS from HICs all report lower prevalence of NDI than was seen in our case cohort, but such studies have several features limiting comparability. A Danish cohort study (1997–2017) found that of 1561 children with a diagnosis of GBS, 3.9% had moderate to severe NDI at 7–18 years of age [32]. However, non-culture-proven GBS cases were included, and International Classification of Diseases, 10th Revision (ICD-10), codes were used for NDI encompassing a range of mental, behavioral, and nervous system disorders, without information on how moderate–severe impairment was defined. A UK population-based cohort study (1998–2017) of 12 533 infants with a diagnosis of GBS found that <1% had cerebral palsy, visual impairment, or hearing impairment [33]. However, it also included non-culture-proven GBS cases, and follow-up was limited to 1 year. Finally, a population-level cohort study from Norway (1996–2019) found, among 866 children with invasive GBS sepsis/meningitis who were followed up to between 20 months and 25 years, that 5.1% developed cerebral palsy, 4.4% epilepsy, 4.2% hearing impairment, 2.5% intellectual disability, and 0.8% visual impairment; however, these were assigned using ICD-10 codes, which may not be consistent [34].

Comparing survivors of GBS sepsis and GBS meningitis, the prevalence of NDI has been reported to be higher after meningitis; 18% had moderate–severe NDI in a 2017 meta-analysis (n = 453) [9]. Although the majority of these studies were from HICs, with only 3/18 from LMICs (China, Tunisia, South Africa), the prevalence remained unchanged when stratified by neonatal mortality rates of ≥5 vs <5 per 1000 live births [9]. A UK study found that 11.8% (6/51) of infant GBS meningitis survivors had cerebral palsy compared with 0/68 GBS sepsis survivors [35].

Adverse growth outcomes in our study were clustered among those with CP and were likely associated with feeding difficulties and dysphagia, rather than iGBS directly. This was also seen in a Ugandan study of infants with neonatal encephalopathy (NE) and a comparison cohort, followed up to 27–30 months of age; undernutrition was significantly more likely in NE survivors than comparators (risk ratio [RR], 2.7); however, this increased risk was driven by those who had NDI (RR, 12.1 comparing those with NDI vs those without NDI, among NE survivors).

### Strengths and Limitations

To our knowledge, this is the first study with 2-year neurodevelopmental outcomes after culture-proven invasive GBS infection among a facility-born cohort in Uganda. With data on comparison children in a 3:1 ratio, we were able to explore differences in characteristics and outcomes of children who had iGBS in early infancy, compared with those who did not. Neurodevelopmental assessment was performed using comprehensive tools and was not dependent on parent report or other subjective measures.

The use of standard BSID-III cutoff scores in this population classified a very high proportion of comparison children as having moderate disability (60%) (Supplementary Data); therefore, we used standard deviations below the mean derived from the comparison cohort to define NDI thresholds. Compared with other cohorts, the prevalence of moderate–severe NDI in our cohort was substantially higher, whether standard or revised NDI cutoff values were used. Two of the 16 in the iGBS cohort were followed up to only 12 months; however, as they had normal neurodevelopmental and growth outcomes at 3, 6, and 12 months, their outcomes would be unlikely to become impaired at 24 months. Gestational ages were not known in over half of iGBS cases (10/16) and were estimated based on birthweight using the Intergrowth 21st charts, which include normative values from children in Sub-Saharan Africa. This may have affected the accuracy of the BSID-III scores, which are based on the corrected age at assessment. However, 8/10 had a birthweight >2.5 kg, indicating that they were term and did not need age correction. For the preterm cases, their scores were not borderline, and therefore classification of NDI was unlikely impacted by the estimated gestations.

Limitations include the size of the cohort, limiting the analysis and interpretation of findings, which meant we were unable to explore risk factors associated with adverse outcomes. Four (19%) participants recruited to the iGBS cohort were lost to follow-up, which led to loss of matching for 4 comparison cohort infants. However, of these, baseline demographic characteristics were not significantly different from those included in the analysis, including gestational age on which cohorts were matched. While this study focused on infants with blood culture–positive GBS sepsis, identifying those with GBS meningitis would have been valuable when considering risk of NDI outcomes; however, in this setting, it was not feasible to perform lumbar puncture for all neonates with suspected sepsis, and therefore not possible to confirm cases of GBS meningitis. Generalizability may be limited by the study sites being 2 tertiary referral hospitals in the capital city of Uganda. Our use of study-specific BSID-II assessment cutoffs for definition of NDI determined from the comparison cohort in this LIC setting may limit generalizability; however, we have also presented results using standardized cutoffs in the Supplementary Data (A and B).

## CONCLUSIONS

In our Ugandan cohort, a high proportion (31%) of survivors of culture-proven iGBS had moderate–severe NDI at 2 years, higher than that reported in other cohorts, whether HIC or LMIC. While interpretation is limited by the small cohort size, our data add to a key evidence gap for neurodevelopmental outcomes after iGBS, particularly from LMICs. Our findings suggest that survivors of iGBS in early infancy should be included in long-term follow-up programs to monitor for evolving developmental disability and initiate timely referrals to support services. In LMICs, improved follow-up pathways and support services for children with NDI are needed.

## Supplementary Data

Supplementary materials are available at *Open Forum Infectious Diseases* online. Consisting of data provided by the authors to benefit the reader, the posted materials are not copyedited and are the sole responsibility of the authors, so questions or comments should be addressed to the corresponding author.

## Supplementary Material

ofae602_Supplementary_Data

## Notes

***Acknowledgments.*** We would like to thank the staff at KNRH, MNRH, and MUCML, as well as all the families and children involved in the study. We would like to recognize our esteemed lab manager, Hannington Tasimwa, who tragically lost his life to COVID-19 during the course of this research. His dedication, expertise, and unwavering support significantly contributed to the success of this project.

The complete PROGRESS Study Author Group is comprised of Abdelmajid Djennad, Agnes Nyamaizi, Agnes Ssali, Alexander Amone, Amusa Wamawobe, Annettee Nakimuli, Caitlin Farley, Carol Nanyunja, Christine Najuka, Cleophas Komugisha, Dan R. Shelley, Edward A. R. Portal, Ellie Duckworth, Emilie Karafillakis, Geraldine O’Hara, Godfrey Matovu, Hannah G. Davies, Janet Seeley, Joseph Peacock, Juliet Nsimire Sendagala, Katie Cowie, Kirsty Le Doare, Konstantinos Karampatsas, Lauren Hookham, Madeleine Cochet, Margaret Sewegaba, Mary Kyohere, Maxensia Owor, Melanie Etti, Merryn Voysey, Moses Musooko, Musa Sekikubo, Owen B. Spiller, Patience Atuhaire, Paul T. Heath, Philippa Musoke, Phiona Nalubega, Pooja Ravji, Richard Katungye, Ritah Namugumya, Rosalin Parks, Rose Azuba, Sam Kipyeko, Simon Beach, Stephen Bentley, Tim Old, Tobius Mutabazi, Valerie Tusubira, and Vicki Chalker.

***Author contributions.*** The research was conceptualized by K.L.D. and C.J.T. C.N. and M.S. collected the clinical data. Data were cleaned and managed by S.S., C.N., V.T., and C.K. Analysis was performed by S.S. with support from C.J.T. S.S. and C.J.T. drafted the manuscript, and all authors contributed to data interpretation, further editing, and critical review of the manuscript. All authors approved the final version.

***Financial support.*** This work was supported by the Bill and Melinda Gates Foundation (OPP1189053). This work was also supported by the National Institute for Healthcare Research (NIHR) for the qualitative research (16/136/46). The content of the manuscript is solely the responsibility of the authors. The funders had no access to the data set of this study and no role in study design, data collection and analysis, decision to publish, or preparation of the manuscript.

***Supplement sponsorship.*** This article appears as part of the supplement “Neonatal and Young Infant Infection in Uganda: the PROGRESS Study,” sponsored by St George's, University of London.

***Patient consent.*** Full written consent was obtained from caregivers for all participants in the study. The study has been approved by the Ugandan National Council for Science and Technology (UNCST; ref. HS 2496), Makerere University School of Medicine Research & Ethics Committee (SOMREC; ref. 2018–130), and St George's, University of London (SGUL REC, ref. 2020.0024), which sponsored the study. The protocol for the PROGRESS study was published in Gates Open Research in June 2020 [12], and the PREPARE study protocol in Wellcome Open Research in August 2022 [13].

***Data availability.*** The full data set for the PROGRESS study is available at doi: 10.24376/rd.sgul.24101115.v1 [36].
