# Supplementary material for: Early Childhood Neurodevelopmental Outcomes After Early Infant Invasive Group B Streptococcal Infection in Uganda
Source: Open Forum Infect Dis. 2025 Mar 10;11(Suppl 3):S173–81. doi: 10.1093/ofid/ofae602 (PMC11891136; doi:10.1093/ofid/ofae602)
Supplement: ofae602_Supplementary_Data [file ofae602_supplementary_data.docx]

# Supplementary information

1. **Neurodevelopmental outcomes using standard BSID-III cut-off scores compared to revised BSID-III cut-off scores, in the GBS sepsis cohort and comparison cohort**

|  | **Standard BSID-III cut-offs** | |  | **Revised BSID-III cut-offs*** | |
| --- | --- | --- | --- | --- | --- |
| **BSID-III score, n (%)** | **Sepsis (n=16)** | **Comparison (n=59)** | **BSID-III score, n (%)** | **Sepsis (n=16)** | **Comparison (n=59)** |
| **Any domain:**  No NDI (≥90)  Mild (85-89)  Moderate (70-84)  Severe (<70)  Mod-Severe (<85)  **Cognitive**:  No NDI (≥90)  Mild (85-89)  Moderate (70-84)  Severe (<70)  Mod-Severe (<85)  **Language**:  No NDI (≥90)  Mild (85-89)  Moderate (70-84)  Severe (<70)  Mod-Severe (<85)  **Motor**:  No NDI (≥90)  Mild (85-89)  Moderate (70-84)  Severe (<70)  Mod-Severe (<85) | 1 (6.3%)  4 (25%)  8 (50%)  3 (18.8%)  11 (68.8%)  5 (31.3%)  1 (6.3%)  7 (43.8%)  3 (18.8%)  10 (62.5%)  2 (12.5%)  4 (25%)  7 (43.8%)  3 (18.8%)  10 (62.5%)  4 (25%)  7 (43.8%)  2 (12.5%)  3 (18.8%)  5 (31.3%) | 6 (10.2%)  18 (30.5%)  35 (59.3%)  0 (0%)  35 (59.3%)  28 (47.5%)  16 (27.1%)  15 (25.4%)  0 (0%)  15 (25.4%)  19 (32.2%)  13 (22%)  27 (45.8%)  0 (0%)  27 (45.8%)  12 (20.3%)  29 (49.2%)  18 (30.5%)  0 (0%)  18 (30.5%) | **Any domain:**  No NDI (>-1SD)  Mild (-1- to -2SD )  Mod (-2- to -3SD )  Severe (<-3SD)  Mod-Severe (<-2SD)  **Cognitive:**  No NDI (≥82)  Mild (77-81)  Moderate (71-76)  Severe (<71)  Mod-Severe (<77)  **Language**:  No NDI (≥81)  Mild (74-80)  Moderate (68-73)  Severe (<68)  Mod-Severe (<74)  **Motor:**  No NDI (≥82)  Mild (78-81)  Moderate (73-77)  Severe (<73)  Mod-Severe (<78) | 5 (31.3%)  5 (31.3%)  1 (6.3%)  5 (31.3%)  6 (37.5%)  6 (37.5%)  5 (31.3%)  1 (6.3%)  4 (25%)  5 (31.3%)  6 (37.5%)  6 (37.5%)  1 (6.3%)  3 (18.8%)  4 (25%)  11 (68.8%)  2 (12.5%)  0 (0%)  3 (18.8%)  3 (18.8%) | 35 (59.3%)  19 (30.5%)  5 (8.5%)  0 (0%)  5 (8.5%)  44 (74.6%)  11 (18.6%)  4 (6.8%)  0 (0%)  4 (6.8%)  45 (76.3%)  14 (23.7%)  0 (0%)  0 (0%)  0 (0%)  55 (93.2%)  3 (5.1%)  1 (1.7%)  0 (0%)  1 (1.7%) |

* Based on standard deviations below the mean in the comparison cohort

**B. Revised BSID-III cut-off scores, compared to standard cut-off scores, based on means and standard deviations in the comparison cohort**

| **Standard BSID-III cut-offs** | | **Revised BSID-III cut-offs*** | |
| --- | --- | --- | --- |
| **Domain** | **Score** | **Domain** | **Score** |
| **Cognitive:**  Mean score  -1 SD  -2 SD  -3 SD  **Language:**  Mean score  -1 SD  -2 SD  -3 SD  **Motor:**  Mean score  -1 SD  -2 SD  -3 SD | 100  85  70  55  100  85  70  55  100  85  70  55 | **Cognitive:**  Mean score  -1 SD  -2 SD  -3 SD  **Language:**  Mean score  -1 SD  -2 SD  -3 SD  **Motor:**  Mean score  -1 SD  -2 SD  -3 SD | 86.1  80.8  75.6  70.3  86.5  80.1  73.6  67.1  85.9  81.7  77.4  73.2 |

* Based on standard deviations below the mean in the comparison cohort
